# Epstein - Barr virus - associated Diseases in Allogeneic Hematopoietic Stem Cell Transplantation

**DOI:** 10.1186/1756-8722-5-S1-A8

**Published:** 2012-04-25

**Authors:** Xiu-li Wu, Qi-fa Liu

**Affiliations:** 1 Department of Hematology, Nanfang Hospital, Southern Medical University, Guangzhou Dadao North Street NO.1838, Guangzhou 510515, Guangdong, China

## 

Epstein - Barr virus (EBV) is a gamma - herpes virus that infects more 90% of humans. Most EBV primary infections and reactivations are subclinical and require no therapy in immunocompetent people. However, EBV infection or reactivation may result in life-threatening diseases in immunocompromised people [1-5]. EBV infection and reactivation might be associated with a spectrum of clinical presentations ranging from fever to post-transplant lymphoproliferative diseases (PTLD), including viremia, pneumonia, encephalitis/ myelitis, PTLD, and so on, in recipients of allogeneic hematopoietic stem cell transplantation (allo-HSCT), which arise as a consequence of infected B/T lymphocytes, epithelial cells and neural cells, and they are sustained by EBV latency products [2,3,6]. EBV-associated PTLD were only the tip of the iceberg of post-transplant EBV-associated diseases [2], but there is short of large sample data about the incidence of EBV-associated other diseases other than PTLD in recipients of transplants. Kinch *et al.* reported that 7 of 16 patients with EBV-DNA-emia developed EBV-associated diseases in 39 recipients of allo-HSCT, including 3 PTLD, 1 myelitis, 1 encephalitis and 2 reactivations with fever [7]. In one of our prospective study, 16 of 64 patients with EBV-DNA-emia developed PTLD and 11 patients developed EBV-associated other diseases, including 6 fever, 1 encephalitis, 1 myelitis, 1 pneumonia, 1 encephalitis accompanying pneumonia and 1 enteritis accompanying hepatitis, in 172 recipients of allo-HSCT. The incidence of PTLD in a large study varied from 0.5% to 22% after allo-HSCT, depending on the number of risk factors including T-depleted graft, the use of ATG, unrelated donor, HLA- mismatched, acute / chronic graft versus host disease (GVHD), cytomegavirus (CMV) antigen-emia, and so on [3,8,9]. Except for lymph nodes, EBV can also involve nearly all other tissues and organs in recipients of transplants, and isolated central nervous system (CNS) involvement with PTLD is considered to be an exceedingly rare complication after allo-HSCT [10]. But our prospective study showed that 16 of 27 patients with EBV-associated diseases were extranodal involvement and 12 patients developed EBV-associated CNS diseases (6 CNS-PTLD, 5 encephalitis and 1 myelitis).

According to the data of the European Group for Blood and Marrow Transplantation (EBMT) [11], RQ-PCR monitoring of EBV-DNA of blood in 73% transplant center has become a routine method for identifying HSCT recipients at risk for developing EBV-associated disease, diagnosis, preemptive therapy and therapeutic evaluation. EBV-DNA loads of blood are acted as the main basis for diagnosis, preemptive therapy and therapeutic evaluation, but our study and others cases reports [12,13] showed that special EBV-associated disease in CNS and pulmonary had the discrepancy in EBV-DNA loads; sometimes repeated testing with real-time PCR in peripheral blood failed to show any evidence of EBV reactivation, and the change of EBV-DNA loads of blood was inconsistent with disease development. But frequent quantitative monitoring of EBV viral load in high-risk patients is still important to prevent occurrence of EBV-PTLD [14].

Important issues of low morbidity and mortality of EBV-associated disease is restoring the immune response to EBV. Therefore, one of prevention and therapeutic option is to manipulate the immune system to target and eradicate these malignancies. In recipients of allo-HSCT in particular, these strategies aim to tilt the balance toward EBV immune responses either by depleting the B-cell population (including EBV-infected B cells) or by augmenting the cellular immune response to EBV. The patients with high risk EBV-DNA-emia are advocated to perform preemptive therapy. Rituximab (anti-CD20) has been applied widely as first-line drug of preemptive therapy. The treatment of EBV-associated diseases include rituximab (AII), reducing immunosuppression (BII), donor lymphocyte infusion (DLI) (CIII) and donor EBV-specific cytotoxic T cells (CTL) infusion (CII), and chemotherapy (CIII). Antiviral agents (EIII) and intravenous immune globulin (IGIV) (DIII) are not recommended for PTLD [5]. Rituximab has dramatically decreased EBV-PTLD incidence and leads to better overall survival [15-18]. The initial response rates of administration of rituximab to EBV-associated diseases ranged from 39.2% to 100% [5,17,19]. But recently report indicated rituximab preemptive treatment was associated with high infection rate and prolonged immune defect [15]. Reducing immunosuppression to restore immune responses to EBV is usually not a useful approach for treating EBV-associated diseases early after allo-HSCT, because the patients are profoundly immunosuppressed and the regenerating immune system usually cannot recover fast enough to eradicate the malignant cells, and meanwhile, high risk of occurrence of GVHD exists. EBV-specific CTL acts as the best treatment of EBV-associated diseases [20], but this approach is currently confined to experimental protocols, and additional drawbacks are the time (2-3 months) and facilities required for CTL production. Other therapeutic strategies such as DLI, chemotherapy, or use of antiviral agents have a limited place in the management of PTLD.

In conclusion, EBV infection or reactivation can present as a variety of clinical symptoms and signs, and involve nearly all tissues and organs in recipients of allo-HSCT. EBV-associated other diseases (other than PTLD), especially CNS and pulmonary diseases, were not rare in recipients of allo-HSCT. EBV-DNA monitoring of blood was a routine method for diagnosis of PTLD and acted as an important indicator for preemptive therapy and therapeutic evaluation. However, case reports demonstrated that patients with isolated EBV-associated CNS PTLD might be the cerebrospinal fluid (CSF) EBV-DNA positive, but blood EBV-DNA negative. The preemptive use of rituximab can reduce the risk of death due to EBV-PTLD in the setting of allo-HSCT. The question of over-treatment can be raised and further studies are needed to select candidates for pre-emptive treatment in order to avoid systematic anti-CD20 treatment and its later potential complications. The problem in developing a treatment for EBV-associated PTLD is the lack of later-phase trials from which to develop evidence-based guidelines. Hopefully, as newer targeted therapies, such as allogeneic EBV-CTL, and more targeted chemotherapy regimens are evaluated, more definitive trials to treat EBV lymphoproliferations will be designed and completed.
